# Sodium bicarbonate intake improves high-intensity intermittent exercise performance in trained young men

**DOI:** 10.1186/s12970-015-0087-6

**Published:** 2015-06-04

**Authors:** Peter Krustrup, Georgios Ermidis, Magni Mohr

**Affiliations:** Department of Nutrition, Exercise and Sports, Section of Human Physiology, Copenhagen Centre for Team Sport and Health, University of Copenhagen, Copenhagen, Denmark; Sport and Health Sciences, College of Life and Environmental Sciences, St. Luke’s Campus, University of Exeter, Exeter, UK; Democrite University of Thrace, Komotini, 69100 Greece; Faculty of Natural and Health Sciences, University of the Faroe Islands, Jónas Broncks gøta 25. 3rd floor, Tórshavn, Faroe Islands; Department of Food and Nutrition, and Sport Science, Center of Health and Human Performance, University of Gothenburg, Gothenburg, Sweden

**Keywords:** Fatigue, Yo-Yo IR2 performance, Blood pH and lactate, Plasma potassium, Buffer capacity

## Abstract

**Background:**

Sodium bicarbonate intake has been shown to improve exercise tolerance, but the effects on high-intensity intermittent exercise are less clear. Thus, the aim of the present study was to determine the effect of sodium bicarbonate intake on Yo-Yo intermittent recovery test level 2 performance in trained young men.

**Method:**

Thirteen men aged 23 ± 1 year (height: 180 ± 2 cm, weight: 78 ± 3 kg; VO_2_max: 61.3 ± 3.3 mlO_2_ · kg^−1^ · min^−1^; means ± SEM) performed the Yo-Yo intermittent recovery test level 2 (Yo-Yo IR2) on two separate occasions in randomized order with (SBC) and without (CON) prior intake of sodium bicarbonate (0.4 g · kg^−1^ body weight). Heart rate and rating of perceived exertion (RPE) were measured during the test and venous blood samples were taken frequently.

**Results:**

Yo-Yo IR2 performance was 14 % higher (P = 0.04) in SBC than in CON (735 ± 61 vs 646 ± 46 m, respectively). Blood pH and bicarbonate were similar between trials at baseline, but higher (P = 0.003) immediately prior to the Yo-Yo IR2 test in SBC than in CON (7.44 ± 0.01 vs 7.32 ± 0.01 and 33.7 ± 3.2 vs 27.3 ± 0.6 mmol · l^−1^, respectively). Blood lactate was 0.9 ± 0.1 and 0.8 ± 0.1 mmol · l^−1^ at baseline and increased to 11.3 ± 1.4 and 9.4 ± 0.8 mmol · l^−1^ at exhaustion in SBC and CON, respectively, being higher (P = 0.03) in SBC. Additionally, peak blood lactate was higher (P = 0.02) in SBC than in CON (11.7 ± 1.2 vs 10.2 ± 0.7 mmol · l^−1^). Blood glucose, plasma K^+^ and Na^+^ were not different between trials. Peak heart rate reached at exhaustion was 197 ± 3 and 195 ± 3 bpm in SBC and CON, respectively, with no difference between conditions. RPE was 7 % lower (P = 0.003) in SBC than in CON after 440 m, but similar at exhaustion (19.3 ± 0.2 and 19.5 ± 0.2).

**Conclusion:**

In conclusion, high-intensity intermittent exercise performance is improved by prior intake of sodium bicarbonate in trained young men, with concomitant elevations in blood alkalosis and peak blood lactate levels, as well as lowered rating of perceived exertion.

## Background

For decades, it has been proposed that muscle acidosis is associated with muscle fatigue during intense exercise [1]. Acidification has been suggested to negatively affect a myriad of steps in the excitation-contraction coupling in the muscle cell, such as activity in the myosin ATPase, Ca^2+^ ATPase and Na^+^-K^+^ ATPase [2], to attenuate the K^+^ efflux through pH-sensitive potassium channels [3, 4] and to impair the activity of metabolic enzymes [1]. During high-intensity intermittent exercise performed in team sports, the high number of intense actions challenges the acid-base homeostasis in the muscle [5, 6] and may, consequently, impair exercise tolerance. Interventions that promote the buffer capacity in the blood and/or muscles may therefore be beneficial in intense intermittent sports.

The Yo-Yo intermittent recovery test level 2 (Yo-Yo IR2) has been demonstrated to have a marked anaerobic component [7]. For example, muscle pH levels below 6.8 have been measured at exhaustion, indicating a high taxation of the acid-base balance in the muscle cell. In addition, Yo-Yo IR2 performance has been shown to correlate to the amount of intense running during the most intense periods in team sport games [8], making the test suitable for studying team-sport-specific exercise with a high anaerobic energy contribution.

Several interventions have been carried out to manipulate the acid-base environment prior to intense exercise, such as drug manipulations [9, 10] and prior arm exercise [11], demonstrating a significant effect on exercise tolerance. For example, β-alanine supplementation has been shown to be beneficial during high-intensity intermittent exercise by increasing the muscle carnosine buffer system [10, 12]. The use of sodium bicarbonate supplementation in order to study fatigue resistance during intense exercise protocols has been widely applied and used by athletes for the last 80 years [13], and a moderate effect size on the influence of the drug on exercise performance has been demonstrated [14]. Bicarbonate doses of 0.3–0.5 g · kg^−1^ body weight have been proposed for inducing consistent performance enhancements in a trained athlete population (for review, see [15]). Some of these studies have also applied team-sports-specific testing protocols [16, 17]. However, to the best of our knowledge no studies have investigated the consequence of sodium bicarbonate supplementation on team-sport-specific exercise that correlates with performance during the most intense periods in competitive match-play and where muscle acidosis has been demonstrated to by high [8].

Thus, the aim of the present study was to examine the effect of oral sodium bicarbonate (0.4 g · kg^−1^ body weight) ingestion on Yo-Yo IR2 performance and physiological response in trained athletes familiar with high-intensity intermittent exercise in training and competition. We hypothesized that sodium bicarbonate intake would result in a more alkaline physiological environment and improve high-intensity intermittent exercise tolerance.

## Methods

### Participants

Thirteen trained male athletes (age: 23 ± 1 year, height: 180 ± 2 cm, weight: 78 ± 3 kg). The athletes had a VO_2_max of 61.3 ± 3.3 mlO_2_ · kg^−1^ · min^−1^ determined by a bicycle ramp-test and were competing in intense sports (middle-distance running, team sports and triathlon) training 5–7 times per week and with a consistent training history of >4 years were recruited to the study. The participants were fully informed of the risks and discomforts associated with the experimental procedures and all provided written consent. The study was approved by the Institutional Research Ethics Committee and conformed to the code of ethics of the Declaration of Helsinki.

### Design

The athletes reported to the laboratory on three separate occasions over a 12-day period. On visit 1, they were familiarized with the Yo-Yo intermittent recovery test level 2 (Yo-Yo IR2; see [7]). The subjects were then assigned in a randomised, crossover design to perform the Yo-Yo IR2 test with (SBC) and without (CON) prior intake of sodium bicarbonate. The test leader was blinded, but no placebo was used in the control trials due to the stomach effects of bicarbonate intake (see [15]). On each experimental visit (2 and 3), the first 2 min of the Yo-Yo IR1 test were completed as a warm-up before the experimental procedures [7]. Venous blood samples were obtained before, during and after the tests as previously described [18], and heart rate (HR) was recorded continuously throughout the experiment (Polar RS400, Polar Electro Oy, Kempele, Finland). Rating of perceived exertion was assessed during the test as described by Borg [19]. The experimental trials were carried out at the same time of day (±1 h). Participants were asked to record their food intake in the 24 h preceding the first experimental trial and to replicate the same diet in the 24 h preceding the subsequent trial. Subjects were instructed to arrive at the laboratory in a rested and fully hydrated state ≥3 h post-prandially and to avoid strenuous exercise in the 24 h preceding each experimental trial. Subjects were also asked to refrain from caffeine and alcohol in the preceding 24 h.

### Experimental procedure

Upon arrival at the laboratory, a cannula (Insyte-W^TM^, Becton Dickinson, Madrid, Spain) was inserted into an antecubital vein to enable frequent blood sampling before, during and after the test. The Yo-Yo IR2 test was performed indoors on a wooden surface on running lanes with a width of 2 m and a length of 20 m. The Yo-Yo IR2 test has been described and evaluated in detail previously [7, 8] and used as an experimental model in other intervention studies on fatigue during high-intensity intermittent exercise [18, 20]. Briefly, it consists of repeated 20-m runs at progressively increased speeds controlled by audio beeps from a CD player. Each running bout is followed by a 10-s active recovery period in which the subject jogs around a marker placed 5 m behind the starting line (for details, see [21]). When a subject twice fails to reach the finishing line in time, the distance covered is recorded and this represents the test result. Blood was sampled at rest (baseline) prior to the Yo-Yo IR2 test, as well as after 160, 280, 440 and 600 m, and at exhaustion in the Yo-Yo IR2 test. In addition, blood was collected at 1, 3 and 5 min of recovery after the test. Blood lactate and glucose, as well as plasma potassium and sodium concentrations, were analyzed in all samples. Blood pH and bicarbonate was analyzed at baseline, immediately before the Yo-Yo IR2 test and at exhaustion.

### Supplementation

After completing the familiarization test, the subjects were assigned in a single-blind, randomized, crossover design to consume sodium bicarbonate (0.4 g · kg^−1^ body weight, SBC) or no supplement (CON). The sodium bicarbonate protocol used in the present study causes a slight stomach discomfort. Therefore the intake was not blinded for the subjects, as also described by others [15]. The sodium bicarbonate was evenly distributed in ~25 gelatin capsules, with one fifth taken at 90, 80, 70, 60 and 50 min prior to exercise, which has been shown to raise blood pH and bicarbonate concentration in pilot experiments.

### Blood and plasma analyses

Blood samples were drawn into 5-mL heparin syringes (Terumo Corporation, Leuven, Belgium). 200 μL of blood was immediately haemolysed in 200 μL of ice-cold Triton X-100 buffer solution (Triton X-100, Amresco, Salon, OH) and analysed to determine blood [lactate] and [glucose] within ~5 min of collection (YSI 2300, Yellow Springs Instruments, Yellow Springs, OH). The remaining whole blood was then centrifuged at 4000 rpm for 3 min (Hetting EBA 20, Tuttlingen, Germany) before plasma was extracted and stored on ice for ~30 min prior to being frozen at -80 **°**C for subsequent analysis of plasma [K^+^] and [Na^+^] (9180 Electrolyte Analyzer, F. Hoffmann-La Roche, Basel, Switzerland). Blood samples for pH and bicarbonate analyses were drawn in 2.5-mL syringes and analysed instantly by an ABL acid-base analyser (Radiometer, Brønshøj, Denmark) [22].

### Rating of perceived exertion (RPE)

RPE was assessed at 160, 280, 440 m and exhaustion according to the 20-stage Borg scale [19].

### Statistical analyses

Differences between SBC and CON in Yo-Yo IR2 performance were analyzed using a paired-samples t-test. Differences between SBC and CON in blood and plasma variables were analyzed using a two-way ANOVA with repeated measures (supplement x time). If a significant F-value was observed, a Tukey post-hoc test was used to identify the points of difference. Statistical significance was accepted at *P* < 0.05. Results are presented as mean ± SEM unless stated otherwise.

## Results

### Performance

**Fig. 1:**
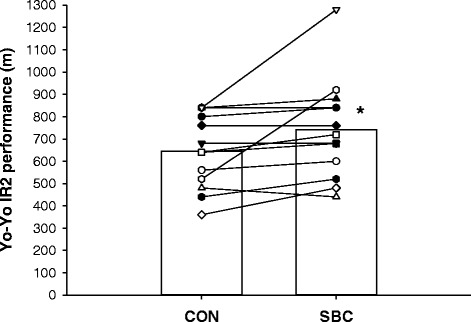
Yo-Yo intermittent recovery test level 2 (Yo-Yo IR2) performance of trained young men (n = 13) on two separate occasions in randomized order with (SBC) and without (CON) prior intake of 0.4 g · kg^−1^ body weight sodium bicarbonate. Individual data and mean data are presented. *: Denotes significant difference between SBC and CON

Yo-Yo IR2 performance was 735 ± 61 m in SBC, which was 14 % higher (P = 0.04) than in CON (646 ± 46 m; Fig. 1). Nine of the thirteen subjects had a better Yo-Yo IR2 performance in the SBC trial, three had the same performance level, and one performed better in the CON trial (Fig. 1).

### Blood and plasma metabolites

**Fig. 2:**
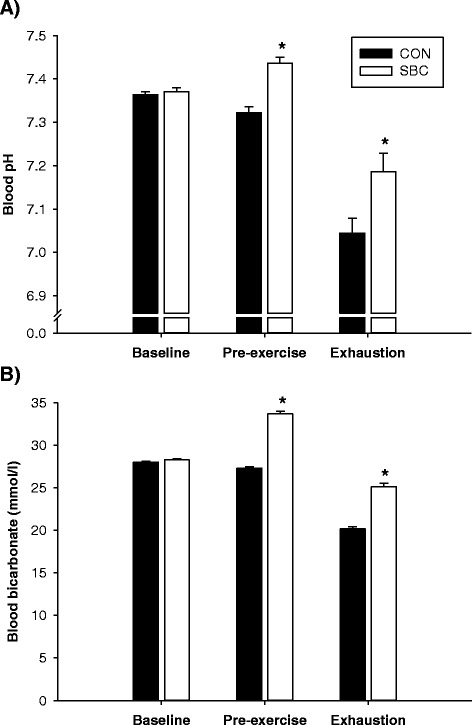
Blood pH (**a**) and blood bicarbonate (**b**) at baseline and immediately before and after the Yo-Yo IR2 test for trained young men (n = 13) on two separate occasions in randomized order with (SBC) and without (CON) prior intake of 0.4 g · kg^−1^ body weight sodium bicarbonate. Data are presented as means ± SEM. *: Denotes significant difference between SBC and CON

Blood pH was 7.37 ± 0.01 at baseline in SBC and CON, but increased (P = 0.002) to 7.44 ± 0.01 in SBC before the Yo-Yo IR2 test and decreased (P = 0.005) in CON (7.32 ± 0.01; Fig. 2a). At exhaustion, blood pH decreased (P < 0.001) to 7.19 ± 0.04 and 7.04 ± 0.03 in SBC and CON, respectively, being higher (P < 0.04) in SBC (Fig. 2a).

Blood [bicarbonate] was 28.3 ± 0.5 and 28.0 ± 0.4 mmol · l^−1^ at baseline, but rose (P < 0.003) in SBC before the Yo-Yo IR2 test (33.7 ± 3.2 mmol · l^−1^) and was unchanged in CON (27.3 ± 0.6 mmol · l^−1^) compared to baseline. At exhaustion, blood [bicarbonate] declined (P < 0.001) in SBC and CON (25.2 ± 2.6 and 20.2 ± 0.8 mmol · l^−1^). Pre-exercise and exhaustive blood [bicarbonate] was higher (P = 0.01 and 0.03) in SBC than in CON (Fig. 2b).

Blood lactate was 0.9 ± 0.1 and 0.8 ± 0.1 mmol · l^−1^ at baseline and increased (P < 0.001) 11.3 ± 1.4 and 9.4 ± 0.8 mmol · l^−1^ at exhaustion in SBC and CON, respectively, being higher (P = 0.03) in SBC (Table 1). Additionally, peak blood lactate was higher (P = 0.02) in SBC than in CON (11.7 ± 1.2 vs 10.2 ± 0.7 mmol · l^−1^).

**Table 1:** Blood and plasma metabolites during the Yo-Yo IR2 test

	Blood lactate (mmol · ^l-1^)		Blood glucose (mmol · ^l-1^)		Plasma K+ (mmol · ^l-1^)		Plasma Na + (mmol · ^l-1^)	
	SBC	CON	SBC	CON	SBC	CON	SBC	CON
Baseline	0.9 ± 0.1	0.8 ± 0.1	4.2 ± 0.2	4.1 ± 0.2	4.3 ± 0.4	4.2 ± 0.4	138 ± 3	138 ± 4
Pre-exercise:	2.0 ± 0.3	1.7 ± 0.2	4.2 ± 0.1	4.3 ± 0.1	4.1 ± 0.4	4.2 ± 0.4	141 ± 4	139 ± 4
Level 17.2:	2.8 ± 0.5	3.0 ± 0.7	4.3 ± 0.1	4.6 ± 0.1	4.8 ± 0.5	4.9 ± 0.5	144 ± 4	140 ± 4
Level 18.3:	4.2 ± 0.8	4.1 ± 0.7	4.5 ± 0.2	4.5 ± 0.2	5.1 ± 0.5	5.2 ± 0,5	143 ± 4	143 ± 4
Level 19.4:	6.3 ± 1.1	6.8 ± 0.9	4.5 ± 0.3	4.8 ± 0.3	5.6 ± 0.6	5.9 ± 0.6	145 ± 4	145 ± 4
Level 20.4:	6.8 ± 1.5	6.2 ± 0.5	4.5 ± 0.2	4.9 ± 0.2	5.1 ± 0.1	5.5 ± 0.2	147 ± 2	144 ± 2
Exhaustion:	11.3 ± 1.4*	9.4 ± 0.8	5.1 ± 0.5	5.2 ± 0.5	5.7 ± 0.5	5.8 ± 0.6	150 ± 4	145 ± 4
1 min recovery:	9.9 ± 1.1	9.0 ± 0.6	5.0 ± 0.5	5.4 ± 0.5	4.4 ± 0.4	4.5 ± 0.4	148 ± 4	145 ± 4
3 min recovery:	10.4 ± 1.1*	9.2 ± 0.6	5.9 ± 0.6	6.0 ± 0.6	3.8 ± 0.4	3.8 ± 0.3	146 ± 4	143 ± 4
5 min recovery:	10.3 ± 1.2*	8.9 ± 0.6	5.8 ± 0.6	5.9 ± 0.6	3.4 ± 0.3	3.7 ± 0.3	145 ± 4	143 ± 3

Blood lactate and glucose as well as plasma K+ and Na + before, during and after a Yo-Yo IR2 performed in randomized order with (SBC) and without (CON) prior intake of sodium bicarbonate. Data are means ± SEM

*Denotes a significant difference from CON. P < 0.05

Blood glucose rose (P < 0.05) from 4.2 ± 0.4 and 4.1 ± 0.2 mmol · l^−1^ at baseline to 5.1 ± 0.5 and 5.2 ± 0.6 mmol · l^−1^ at exhaustion, with no difference between trials (Table 1).

Plasma [K^+^] rose (P < 0.05) from 4.3 ± 0.4 and 4.2 ± 0.4 mmol · l^−1^ at baseline to 5.7 ± 0.5 and 5.8 ± 0.6 mmol · l^−1^ at exhaustion in SBC and CON, respectively, with no differences between trials (Table 1). Plasma [Na^+^] was unchanged during the test and not different between conditions (Table 1).

### Heart rate and rating of perceived exertion

Heart rate was 78 ± 2 and 79 ± 2 bpm at baseline and 91 ± 3 bpm pre exercise in SBC and CON, respectively. Heart rate increased (P < 0.05) during the test, reaching 197 ± 3 and 195 ± 3 bpm at exhaustion in SBC and CON, respectively. No difference in heart rate response between conditions was determined during or after the test.

RPE was not different between trials after 160 and 280 m (SBC: 13.0 ± 0.7 and 14.8 ± 0.7; CON: 13.1 ± 0.7 and 15.0 ± 0.6, respectively), but was 7 % lower (P = 0.003) after 440 m in SBC than in CON (16.8 ± 0.4 vs 17.9 ± 0.3). At exhaustion, RPE was similar in SBC and CON (19.3 ± 0.2 and 19.5 ± 0.2).

## Discussion

In the present study, we observed that prior intake of sodium bicarbonate in capsular form using a protocol with gradual intake enhanced high-intensity intermittent exercise performance in young trained males. The performance improvement after sodium bicarbonate ingestion was accompanied by an elevated blood alkalosis and concentration of bicarbonate. In addition, blood lactate concentrations at exhaustion and peak values reached during the experimental protocol were higher, while the rating of perceived exertion was lower during intense exercise after sodium bicarbonate supplementation. In contrast, blood glucose, plasma K^+^ and Na^+^ as well as cardiovascular loading during high-intensity intermittent exercise were unaffected by sodium bicarbonate intake.

Performance in the Yo-Yo IR2 test increased by 14 % after sodium bicarbonate intake, which is comparable to a 16 % increase after caffeine intake in a comparable athlete population [18], but lower than a 34 % improvement after β-alanine supplementation in football players [23]. In comparison, training studies report a 19–29 % improvement in Yo-Yo IR2 performance observed after short-term periods (4–8 weeks) with speed endurance training in moderately trained [20] and well trained males [24]. Support for the performance improvement in the present study for the sodium bicarbonate intervention is provided, for example, by enhanced performance in the final sprints of a repeated sprint test [16, 25] and simulated boxing performance [17] after sodium bicarbonate intake, indicating an ergogenic effect during high-intensity exercise conditions performed under metabolic stress comparable to the short, intermittent and anaerobic Yo-Yo IR2 exercise protocol [7, 8, 21]. However, some studies applying intense exercise regimes such as Wingate testing report no beneficial effect of sodium bicarbonate supplementation (for review, see [14, 15]), indicating a predominant effect of sodium bicarbonate on high-intensity intermittent exercise. According to a review by Carr et al. [15], sodium bicarbonate has a greater effect on trained individuals, so differences in training status may also partly explain the discrepancy between findings in different studies. In the present study, the subjects were well trained, having a maximal oxygen uptake comparable to top-class team-sport athletes and a Yo-Yo IR2 performance slightly below top-class central defenders in football [21]. Moreover, they were all involved in high-intensity sport, and the results indicate that this athlete population may benefit from sodium bicarbonate intake.

In the present study, we did not apply a placebo, since the gastrointestinal effects of high doses of bicarbonate are usually easy to trace [15]. A potential placebo effect may therefore have had an impact on the results, which is a limitation of the study design. However, in the present study a 14 % improvement was seen in the sodium bicarbonate trial, with nine of the participants displaying an improvement and only one having a decline in performance. In comparison, the placebo effect on high-intensity exercise performance reported in the literature is 1–3 % [26]. There are two subjects that display a very large improvement in performance (~50 and ~75 %; Fig. 1) and are likely to be high-responders to the sodium bicarbonate, which may have affected the results. However, even when these two outliers are omitted from the statistical analysis, there is still a significant 6 % increase in Yo-Yo IR2 performance after sodium bicarbonate intake.

Part of the improvement in intermittent exercise performance may be explained by an elevated bicarbonate-induced buffer capacity in the blood, which will increase the muscle-to-blood H^+^ gradient [27]. Before the start of the Yo-Yo IR2 test, blood bicarbonate was elevated by 23 % in the sodium bicarbonate trial compared to control, with a significantly increased blood pH value. Thus, the blood buffer capacity was increased, which also resulted in higher blood pH and bicarbonate concentrations at exhaustions, despite a markedly longer exercise time in the intervention trial. The anaerobic contribution to the energy yield also appeared to be higher after the treatment with sodium bicarbonate, as reflected by the 15 and 20 % higher peak blood lactate and exhaustion levels, which may also be associated with longer exercise time.

Fatigue development during high-intensity intermittent exercise may be caused by a complex interplay between intra- and extracellular concentrations and gradients of ions such as K^+^, Na^+^, Cl^−^ and H^+^ [28, 29]. In the present study, no differences were detected in plasma [K^+^] and [Na^+^] between the sodium bicarbonate and control trials. However, since the potential fatiguing effect of a homeostatic imbalance in these ions is exerted in the muscle interstitial compared to intracellular environment [28–32], a sodium bicarbonate induced effect on the kinetics of these ions during exercise cannot be ruled out. This is especially relevant to consider when the venous ion concentrations do not seem to reflect the interstitial values during intense exercise [30]. In support of a link between alkalosis and improved muscle K^+^ regulation, Street et al. [27] found, using the microdialysis technique in human skeletal muscle, that the interstitial K^+^ accumulation rate during intense exercise was attenuated after drug-induced alkalosis.

In the present study, RPE was lowered after 440 m of running in the Yo-Yo IR2 test in the sodium bicarbonate trial compared to the control, despite the fact that the heart rate and blood lactate concentration at this time-point were similar between trials. This may suggest that centrally mediated mechanisms were affected. The participants experienced less exertion late in the Yo-Yo IR2 test in the SBC trial when the heart rate was >95 % of HR_max_ and blood lactate and plasma K^+^ were markedly elevated. Moreover, although further distance was covered before exhaustion, the RPE scores remained the same as in the control trial at the point of fatigue. Thus, a higher performance level was achieved while reporting an equal level of perceived fatigue at exhaustion. Peripheral alterations are likely to lead to modulation of neural strategies, for example via group III and IV muscle afferents, widely distributed through muscle and responsive to a variety of chemical stimuli, including altered H^+^ [33, 34], which was lower in the present study after sodium bicarbonate intake. Thus, part of the improved performance after sodium bicarbonate treatment may relate to less negative feedback from the muscle and, thereby, less effect on the descending drive to the motoneurons [33, 35].

## Conclusions

High-dose sodium bicarbonate intake (0.4 g · kg^−1^ body weight) improved high-intensity intermittent exercise performance in trained young men, with concomitant increased blood alkalosis. Sodium bicarbonate supplementation increased blood lactate levels at exhaustion and lowered rating of perceived exertion during intense intermittent exercise. The results indicate a link between improved fatigue resistance during high-intensity intermittent exercise and a sodium-bicarbonate-induced improved buffer capacity that may affect perceived exertion during intense intermittent exhaustive exercise.
